# The Role of Endoscopic Ultrasound in the Diagnosis and Management of Primary Gastric Lymphoma

**DOI:** 10.1155/2017/2397430

**Published:** 2017-03-16

**Authors:** Dimitrios Schizas, Ioannis Ntanasis-Stathopoulos, Diamantis I. Tsilimigras, Athanasios D. Sioulas, Demetrios Moris, Eleftherios Spartalis, Ilias Scotiniotis, Ioannis S. Papanikolaou

**Affiliations:** ^1^First Department of Surgery, Laikon General Hospital, National and Kapodistrian University of Athens, Athens, Greece; ^2^School of Medicine, National and Kapodistrian University of Athens, Athens, Greece; ^3^Department of Gastroenterology, Hygeia Hospital, Athens, Greece; ^4^Department of Immunology, Lerner Research Institute, Cleveland Clinic, Cleveland, OH, USA; ^5^Second Department of Propaedeutic Surgery, National and Kapodistrian University of Athens, Athens, Greece; ^6^Hepatogastroenterology Unit, Second Department of Internal Medicine and Research Unit, “Attikon” University General Hospital and National and Kapodistrian University of Athens, Haidari, Greece

## Abstract

Endoscopic ultrasound (EUS) is considered a valuable diagnostic tool during the workup of malignant gastric lesions, including primary gastric lymphomas (PGL). Although endoscopy combined with multiple biopsies remains essential in the establishment of PGL diagnosis, EUS utilization in locoregional disease staging has been well documented in the literature. Data also support the possible role of EUS in prediction of response to first-line treatment, that is, *Helicobacter pylori* eradication. However, its application in the posttreatment setting remains problematic, since concordance rates between endosonography and histology findings during follow-up seem to vary substantially. The aim of the present review is to summarize all available data regarding the role of EUS in the management of PGL.

## 1. Introduction

Non-Hodgkin lymphoma (NHL) consists of a diverse group of malignancies originating from the lymphoid tissue and deriving from the clonal expansion of B-cells, T-cells, natural killer (NK) cells, or their precursors. Most NHL present with disease located in lymph nodes, which comprise about 65–80% of all cases [1, 2]. On the other hand, primary extranodal NHL, by definition, involves organs or tissues other than lymph nodes or spleen. Amongst them, gastrointestinal lymphoma accounts for the 30–40% of cases. The most frequently involved site is the stomach (60–75%) followed by the small bowel, ileum, cecum, colon, and rectum [1, 3]. The vast majority (greater than 90%) of primary gastric lymphomas (PGL) are equally divided into two major histologic subtypes: mucosa-associated lymphoid tissue (MALT) and diffuse large B-cell lymphoma (DLBCL). The remaining cases include mantle cell (1%), follicular cell (0.5–2%), and peripheral T-cell lymphomas (1.5–4%) [4–6].

Gastric MALT lymphoma comprises about 50% of PGL. Although a strong association with chronic *Helicobacter pylori* infection had been initially demonstrated [7], recent data have shown that the incidence of *H. pylori*-positive lymphomas has remarkably declined during the last decades reaching to 35% approximately [8]. In these cases that are unlikely to respond to *H. pylori* eradication treatment, there are additional therapeutic options with favorable efficacy including radiotherapy, chemotherapy, and immunotherapy [9, 10]. Current treatment strategies take into account both the presence of *H. pylori* and the stage of the disease. It has to be noted that several staging systems have been proposed throughout the years (Table 1) [11–13]. In that context, endoscopic ultrasound (EUS) of the stomach has emerged as one of the best tools for locoregional staging in PGL. Indeed, several studies have demonstrated its high accuracy in the initial staging and, possibly, the prediction of response to treatment and posttreatment follow-up [14, 15].

In this review, we aimed to present the available data regarding the role of EUS in PGL both in the diagnostic and posttreatment settings.

### 1.1. Diagnosis and Locoregional Staging

**Figure 1:**
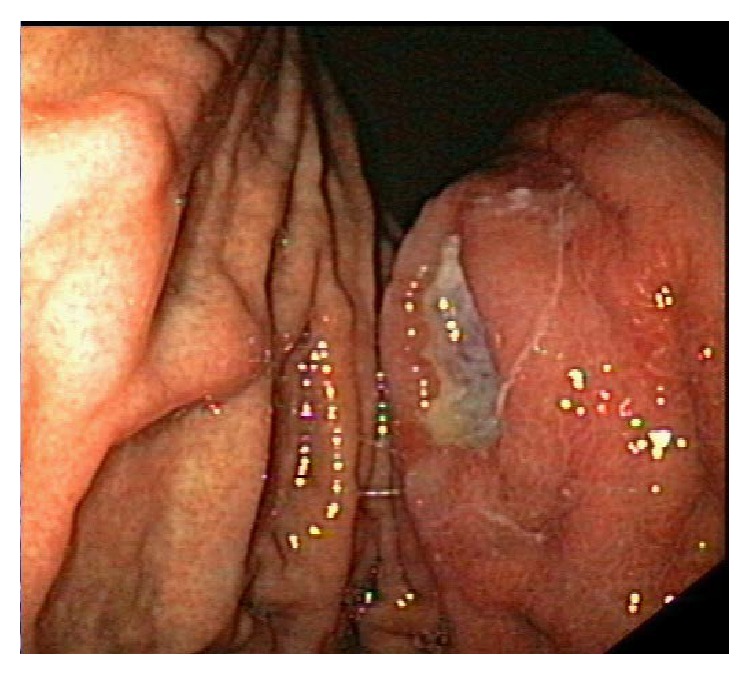
Endoscopic image of primary gastric lymphoma.

**Figure 2:**
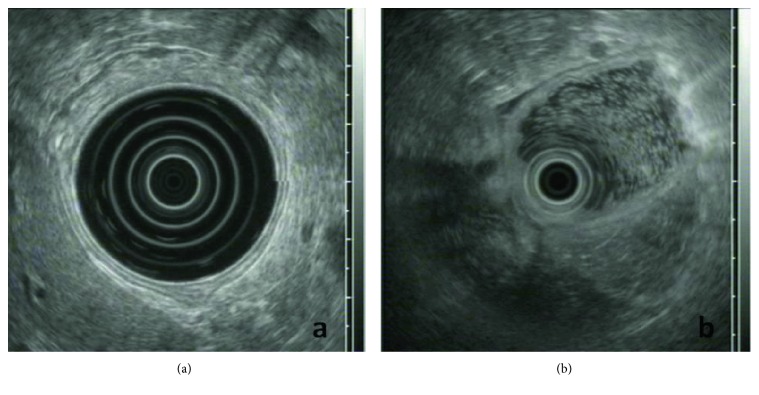
(a) Radial-EUS imaging of the gastric wall; notice the normal 5-layer structure. (b) Radial-EUS imaging in primary gastric lymphoma; notice the disappearance of the normal 5-layer structure at the point where the EUS transducer comes into contact with the gastric wall (9 to 6 o'clock position).

Patients suffering from PGL usually present with nonspecific upper gastrointestinal (GI) complaints, such as vague abdominal pain, melena, and hematemesis that eventually lead to an extensive diagnostic workup including endoscopic series. Endoscopy may not detect the neoplastic lesion, as it can develop in deeper GI layers; however, when combined with multiple biopsies taken from different sites, such as the stomach, gastroesophageal junction, duodenum, and abnormal-appearing areas, its efficacy in the diagnostic procedure significantly increases [16]. As the endoscopic appearance of a PGL varies from normal gastric pattern or subtle mucosal irregularities to large ulcers, a high index of suspicion is rendered crucial in order to reach an accurate diagnosis (Figure 1). Accordingly, endosonographic features of PGL may either be initially invisible or vary from a thickening of the inner two or three layers to a diffuse wall thickening, with or without preservation of the typical 5-layer structure (even if the layers are thickened or distorted, with an irregular, but relatively well-preserved outer margin) (Figures 2(a) and 2(b)) [5, 6].

Furthermore, it has to be highlighted that EUS pattern may correlate with the histologic subtype of PGL. Indeed, Suekane et al. showed that superficial spreading or diffuse-infiltrating lesions on EUS were associated with MALT lymphoma, while mass-forming lesions were associated with gastric DLBCL [17]. Given the small cohort of patients included in this study, however, this evidence cannot directly be applied in clinical practice. In general, endoscopy combined with multiple biopsies is considered—to date—the gold standard in diagnosing an abnormal-appearing lesion, which is essential for determining the appropriate treatment [18].

Currently, EUS is considered the method of choice for locoregional staging of PGL, including the detection of affected perigastric lymph nodes. It has been shown that EUS is superior to CT scan in this setting due to higher sensitivity both in detecting lymph node involvement as well as subtle differences regarding gastric layers and wall thickness [19]. This is of great importance since locoregional staging is one of the major factors that can predict the response to treatment, that is, the identification of patients whose disease is likely to be refractory to treatment or to recur.

Another aspect of EUS staging in PGL is the evaluation of lymph node involvement. Regional lymph nodes as well as those beyond the regional area may be demonstrated with EUS. Their characterization as affected or nonaffected by lymphoma is initially decided according to the classical B-mode criteria indicating malignancy, namely, hypoechoic structure, sharply demarcated borders, rounded contour, and size > 1 cm. These criteria were established in the 1990s for esophageal carcinoma, but their use has been widely extrapolated for any type of malignancy including prediction of lymph node infiltration [20].

Although these criteria were extremely useful at the time they were set, modern noninvasive improvements including contrast enhancement and elastography have been introduced in an attempt to improve the accuracy of EUS for N staging. In a study from Germany, contrast-enhanced EUS showed improvement in diagnosing benign lymph nodes compared to standard EUS, but it did not increase the accuracy in detecting malignant lymph nodes [21]. Additionally, elastography is a technique that offers information on mechanical properties of the examined tissue by measuring mechanically induced deformations (i.e., strain) of structures in B-mode images in order to quantify the elasticity of the tissue. It was introduced in EUS imaging as a promising approach, since it could enable a noninvasive method in the N staging, based on the fact that malignant lymph nodes are generally “harder” than their nonmalignant counterparts [22]. This technique has been validated for metastatic carcinomas [23], as well as primary carcinomas per se [24], increasing the accuracy of EUS in detecting malignant lymph nodes to 85%. However, data regarding its use in PGL is still limited. Although the advent of up-to-date technology in EUS instruments is highly appreciated, the limited available data from the literature prevent its wide application in everyday clinical practice.

Literature from the early 1990s had determined the accuracy of EUS for PGL T and N staging at a level of approximately 90% and 80%, respectively [14, 15]. However, in 2002, a multicenter study incorporating data from 34 centers (including 70 patients) showed that the overall accuracy of EUS in determining the stage (according to modified Ann Arbor classification) was 53%. Nevertheless, it has to be noted that the majority of examiners in most centers were probably not experienced in PGL staging, as indicated by the fact that only five out of 34 centers recruited more than 2 patients in the study [25]. Although this study may falsely underestimate the role of EUS in locoregional staging of PGL, it highlights the well-known problem of operator's expertise during EUS staging. According to recent guidelines, improvement in EUS training along with recent advances in EUS technology, namely, electronic instead of mechanical EUS imaging, Doppler ultrasound, elastography, and lately the use of intravenous contrast medium for EUS, could possibly increase the efficacy of this modality, even in the hands of less experienced examiners [26]. Another intriguing issue affecting the quality of EUS staging is the reproducibility of its results; in a study examining interobserver agreement, the authors concluded that it was at an acceptable level for T staging (kappa: 0.38) and substantial for N staging (kappa: 0.63), whereas the lowest values of agreement were detected for T1sm (submucosa) and T2 stage lesions (kappa: 0.33–0.35) [27]. However, it must be stressed that these results come from a center with specialists in the field and these do not always reflect the daily clinical practice.

Overall, two main factors seem to strongly influence the reproducibility of EUS findings: training and experience. Apart from the above, it has to be underlined that regular good quality endoscopic biopsy sampling is of utmost importance. In the era of molecular characterization of lymphomas that is associated with predictive and prognostic aspects, it is very important to assure the availability of a diagnostic material [28].

### 1.2. Is There a Role for EUS-FNA?

Several years ago, the advent of EUS-guided fine needle aspiration (FNA) biopsy provided new possibilities for transmural tissue diagnosis. This technique allows for cytological examination of the specimen, in contrast to the endoscopic biopsy that refers to histological evaluation. Although EUS-FNA seems to be efficient in mediastinal and intra-abdominal malignancies [29–31], its exact role in the staging of PGL has not been formally investigated and is highly debatable. Nevertheless, two studies have demonstrated its importance in the diagnosis of nodal lymphoma; the first revealed the high diagnostic accuracy of EUS-FNA when combined with flow cytometry and immunocytochemistry, but it was limited by its retrospective nature and the rather small number of patients (i.e., 38, 23 with lymphoma, and 15 patients with benign disease or reactive lymphadenopathy) [32]. The second was a Japanese study involving a total of 104 patients with mediastinal and/or intra-abdominal lymphadenopathy of unknown origin that demonstrated a high accuracy for EUS-FNA in achieving the appropriate diagnosis (48/50 lymphomas, i.e., 96%) and a similar ability to correctly classify the lymphomas in 44/50 of cases (88%) [29]. Though not explicitly reporting on PGL, both studies indicate that EUS-FNA could aid in determining nodal involvement in this setting.

There are, however, limitations owing to the intrinsic properties of this technique. There remain concerns that only a limited amount of tissue can be removed with EUS-FNA, thus reducing its diagnostic ability. This could be overcome by using needles with a larger caliber (e.g., 19-gauge), which allow tissue acquisition, histologic evaluation, and subsequent classification of lymphoid tissue malignancies. Furthermore, it has been shown that subclassification of lymphomas solely by EUS-FNA is feasible only in 66% of cases yielding the lowest accuracy regarding low-grade lymphomas. The proper distinction between benign hyperplasia and low-grade lymphomas based solely on morphological or immunohistochemical features has been questioned [33]. Additionally, it is difficult during EUS to determine the most appropriate lymph nodes to perform sampling in low-grade lymphomas, due to the fact that echogenic properties in these cases are of limited value and only size may be used as a guide. In contrast to these restrictions, cytological diagnosis is more feasible in high-grade lymphomas/DLBCL [33]. Lastly, there is yet no comparative study examining EUS-FNA with the standard histologic evaluation, in order to clearly define the superiority or inferiority of this approach.

### 1.3. Prediction of Response to Treatment with EUS

Current guidelines favor the administration of *H. pylori* eradication regimens as the first-line treatment in *H. pylori*-positive patients irrespective of tumor stage [9, 10]. As aforementioned, locoregional staging is associated with the prediction of response to treatment and, therefore, EUS may provide valuable prognostic information in this setting. Regarding MALT lymphoma, EUS-based staging confers prognostic value as localized disease seems to respond well to *H. pylori* eradication treatment, while advanced disease shows greater resistance to therapy [10, 34, 35]. However, there are some reports of complete disease regression after *H. pylori* eradication in lymphomas of even more advanced stages [32]. It should be noted that the complete regressions occurring in these cases (3/5 patients with stage EII gastric MALT lymphomas) could be a result of overstaging due to reactive inflammation that led to echo-poor wall infiltration beyond the limits of the lymphoid tissue or due to reactive enlargement of lymph nodes [32]. It has also to be underscored that PGL staging might be of higher value in DLBCL subtype. EUS staging along with molecular biomarkers may be the basis for determining the probability of response to *H. pylori* eradication treatment solely, before proceeding to systemic therapy that is usually necessary in patients with DLBCL [36].

### 1.4. The Role of EUS in the Posttreatment Follow-Up

Data regarding the role of EUS in the follow-up of gastric lymphoma after treatment seem to be controversial. Initial studies from the time when chemotherapy was an option even for early stage low-grade gastric MALT lymphomas showed that EUS is a reliable method in assessing response to chemotherapy [37]. However, more recent publications showed that an echo-poor infiltration of the gastric layers might persist for more than 6 months despite complete disease remission; therefore, persistence of an EUS abnormality in the gastric wall structure with a negative histology should not imply evidence of disease requiring further treatment per se. Interestingly, the endosonographic appearance of the wall structure may appear normal despite the presence of lymphoma resistant to therapy. At least two well-conducted studies have confirmed these findings by showing that standard endoscopic biopsies were superior to EUS in the monitoring of patients with PGL [38, 39]. It should be also mentioned that the concordance rates between EUS and histological findings during the follow-up period vary substantially in the literature, and, thus, concerns are raised [19]. Due to the inferior accuracy rates reported for EUS compared to histology, repeated upper GI endoscopy with biopsies every 6 months for the first 2 years and then annually is considered sufficient for the follow-up of patients with PGL [40, 41].

## 2. Conclusion

EUS constitutes a valuable diagnostic tool during the staging workup of PGL, which has shown superiority over the CT scan regarding the locoregional disease assessment. In this setting, EUS may provide important information on prognostic features of the disease and may contribute to the determination of the therapeutic approach. To date, its role in the assessment of disease response to treatment remains rather limited and controversial. However, taken into consideration the vivid interest of the scientific community on this issue, future multicenter collaborative studies are necessary in order to shed light into the role of EUS in the management of PGL.

## Conflicts of Interest

The authors declare that there is no conflict of interest regarding the publication of this paper.

## Figures and Tables

**Table 1:** Comparison of Ann Arbor, Lugano, and Paris (TNM) classification systems.

Ann Arbor staging system		Lugano classification	Paris classification (TNM)	Lymphoma extension
IE1	Confined to mucosa, submucosa	Stage I	T1–T3N0M0	Confined to GI tract (mucosa, submucosa, muscularis propria, and serosa)
IE2	Confined to the stomach, invasion of the muscularis and/or serosa			

IIE1	Involvement of the stomach and contiguous lymph nodes	Stage II II1: local nodal involvement II2: distant nodal involvement	T1–T3N1M0 T1–T3N2M0	Extending into the abdomen
IIE2	Involvement of the stomach and noncontiguous subdiaphragmatic lymph nodes			Perigastric lymph nodes
				More distant regional lymph nodes

IIIE	Involvement of the stomach and lymph nodes on both sides of the diaphragm	Stage III	T4N0M0	Penetrating of serosa and adjacent organs
			T1–T4N3M0	Lymph nodes on both sides of the diaphragm

IVE	Hematogenous spread	Stage IV	T1–T4N0–N3M1	Distant metastasis (e.g., bone marrow)
